# Trifecta of Tumors: Simultaneous Detection of Three Primary Malignancies by Different Radiotracers of Nuclear Medicine

**DOI:** 10.1055/s-0044-1788738

**Published:** 2024-07-24

**Authors:** Siven Kar, Harshita Gupta, Nusrat Shaikh, Vikram Lele

**Affiliations:** 1 Department of Nuclear Medicine and PET-CT, Jaslok Hospital and Research Centre, Mumbai, Maharashtra, India

**Keywords:** multiple primary malignancies, PSMA, FDG, PET, thyroid carcinoma.

## Abstract

Malignancies are increasing worldwide with changing lifestyle, pollution, increasing life expectancy, and diagnostic advancements. However, multiple primary malignancies (MPMs) detected simultaneously are very rare. Here, we present a rare case of three primary malignancies (sigmoid colon, prostate, and thyroid) detected simultaneously in a 77-year-old male patient, who initially presented with bleeding per rectum and was then found to have a large pedunculated mass in the sigmoid colon on colonoscopy, which further turned out to be adenocarcinoma. On further imaging and investigations, two new separate malignancies (prostate and thyroid) were found by two different positron emission tomography radiotracers: prostate-specific membrane antigen (PSMA) and fluorodeoxyglucose (FDG). Hence, nuclear medicine modalities can play an important role in detecting MPMs using the vast array of radiotracers available now and perhaps reduce the need for multiple biopsies.

## Introduction

Multiple primary malignancies (MPMs) are rare. They are defined as more than one primary malignancy, which are histologically unrelated. Triple primary malignancies are even rarer and their incidence has been reported to be between 0.04 and 0.81%. 1 Early detection and treatment are very important in the clinical management of such patients. Here, we report a case with three different primary malignancies detected simultaneously over a period of less than 3 months using two different radiotracers of nuclear medicine— ^68^ Ga-labeled prostate-specific membrane antigen positron emission tomography/computed tomography ( ^68^ Ga-PSMA PET/CT) and ^18^ F-fluorodeoxyglucose PET/CT ( ^18^ F-FDG PET/CT). Also, surprisingly sigmoid colon cancers, which are known to be PSMA avid, did not show any PSMA uptake on our scan. 2

## Case History

**Fig. 1:**
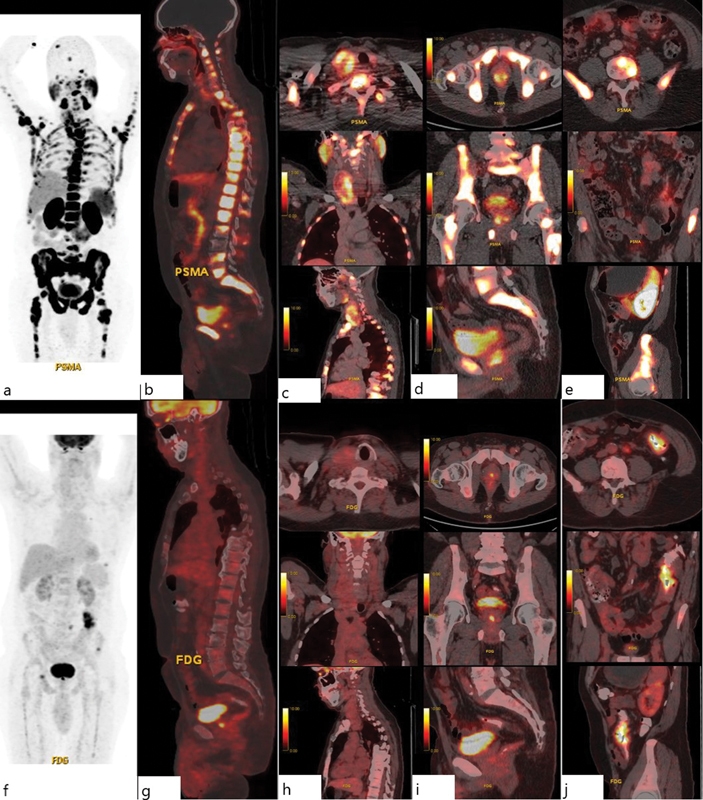
^68^ Ga-labeled prostate-specific membrane antigen ( ^68^ Ga-PSMA) positron emission tomography (PET)/computed tomography (CT): ( **a** ) Maximum intensity projection (MIP) image. ( **b** ) Sagittal image showing intense PSMA expression in extensive osteosclerotic lesions. Axial, coronal, and sagittal images showing intense PSMA expression in the heterogeneously enhancing lesion in the ( **c** ) right thyroid gland, ( **d** ) prostate, and ( **e** ) low-grade PSMA expression in concentric wall thickening of the sigmoid colon. Fluorine-18 fluorodeoxyglucose ( ^18^ F-FDG) PET/CT: ( **f** ) MIP image. ( **g** ) Sagittal image showing low metabolic activity in the extensive osteosclerotic lesions. Axial, coronal, and sagittal images showing low metabolic activity in the lesion in the ( **h** ) right thyroid gland, ( **i** ) prostate, and ( **j** ) hypermetabolic concentric wall thickening of the sigmoid colon.

A 77-year-old man presented with complaints of difficulty in passing stools and bleeding per rectum. Colonoscopy revealed a large pedunculated tumor in the sigmoid colon. Biopsy revealed an invasive moderately differentiated adenocarcinoma of the sigmoid colon. He was referred for ^18^ F-FDG PET/CT. It showed hypermetabolic circumferential wall thickening of the sigmoid colon with a hypermetabolic pericolic lymph node. There was incidental detection of extensive osseous sclerotic skeletal lesions, solitary pulmonary nodule in the left upper lung, and heterogeneous lesion in the right lobe of the thyroid, all of which showed mildly increased metabolic activity. Prostatomegaly was also noted; however, no abnormal tracer uptake was seen in the prostate. Serum PSA was found to be elevated (376 ng/mL). ^68^ Ga-PSMA PET/CT showed diffuse intensely increased PSMA expression in the enlarged prostate and extensive sclerotic skeletal lesions—T4N0M1b ( Fig. 1 ). The heterogeneously enhancing circumferential wall thickening of the sigmoid colon showed no PSMA expression. There was, however, high-grade PSMA expression in the heterogeneously enhancing lesion with central hypodense areas in the right lobe of the thyroid—a third primary malignancy was suspected, for which we had advised a histopathological correlation. No PSMA expression was noted in the pulmonary nodule in the left upper lung. Tru-cut biopsy from the prostate revealed adenocarcinoma (Gleason's score if 3 + 4 = 7). The patient underwent right hemithyroidectomy (biopsy revealed low-grade follicular carcinoma—T3aN0M0) and exploratory laparotomy with resection of the sigmoid colon with a side-to-side colorectal anastomosis on the same day (T3N1M0). Postoperative stay was uneventful. He was planned for four cycles of ^177^ Lu-PSMA radioligand therapy.

## Discussion

MPMs are described as the presence of more than one primary malignancy, which are histologically unrelated, in an individual. Warren and Gates' criteria have been widely used to define MPMs, which include (1) histological confirmation of each malignancy, (2) tumors separated by at least 2 cm of the normal mucosa, and (3) probability of one being metastatic to the other has to be excluded. They are further divided as synchronous or metachronous. The frequency of these MPMs, according to previous epidemiologic studies (depending on the criteria used), varies from 2.4 to 17%. 3 According to Surveillance, Epidemiology, and End Results (SEER) database, synchronous malignancies are those that are diagnosed within a time interval of 2 months and metachronous malignancies are those diagnosed more than 2 months after the diagnosis of the first primary cancer, whereas the International Agency for Research on Cancer (IARC) recommends a time interval of 6 months. 3 They have also been classified by Moertel into group I, which includes cancers arising from organs with the same histology; group II, which includes cancers arising from different tissues; and group III, which includes cancers from different tissues and organs that concurrently exist with the group I cancers. 4

The prevalence of both synchronous and metachronous malignancies has increased with the aging population, increased exposure to carcinogens, lifestyle changes, environmental factors, long-term side effects of radiotherapy and chemotherapy, etc. 5 This makes long-term follow-up very important in treated cancer patients.

With increasing use of whole-body scans like ^18^ F-FDG PET/CT, occasional detection of new primary lesions, which were missed by standard CT, is not uncommon. In a series involving 1,912 patients by Ishimori et al, ^18^ F-FDG PET/CT detected additional suspected new primary lesions in 4.1% of the patients. However, only 1.2% turned out to be malignant. 6

Second primary malignancies are associated with multiple familial cancer syndromes like Lynch's syndrome, von Hippel–Lindau (VHL) syndrome, Cowden's syndrome, Li–Fraumeni syndrome, Hereditary Breast and Ovarian syndrome, etc.

PSMA is a type II transmembrane metallopeptidase glycoprotein that is highly expressed in prostate cancer. It has also been found to be overexpressed in the neo-vasculature of other nonprostate solid tumors. These include thyroid, non-small-cell lung carcinoma, renal cell carcinoma, breast cancer, gastric and colorectal cancers, etc. These could have potential future therapeutic applications. According to Haffner et al, PSMA expression was detected in the neo-vasculature of 85% of colorectal carcinomas. 2 However, there was no PSMA expression in the sigmoid colon cancer in our patient.

There has been a similar case report with dual primary malignancy of the prostate and colorectum, which was imaged with both FDG and PSMA. 7 ^18^ F-FDG PET/CT showed uptake in colorectal malignancy with heterogeneous uptake in the enlarged prostate. Following this, ^68^ Ga-PSMA PET/CT was done, which showed prostate malignancy with bone metastasis. However, there was no significant PSMA expression in the colorectal malignancy.

^68^ Ga-PSMA uptake is known in malignant differentiated thyroid cancers (DTCs), which has been confirmed by immunohistochemical assessment. PSMA expression has been correlated with tumor size, vascular invasion, radioiodine refractoriness, poor prognosis, and poor progression-free survival (PFS). 8 9 The anaplastic thyroid carcinoma (TC) shows lower PSMA expression compared to the differentiated type despite its aggressiveness, likely secondary to its lower microvessel density. However, compared to ^18^ F-FDG PET/CT, PSMA PET detected fewer lesions and hence has not been able to replace it as the hybrid imaging of choice for radioiodine refractory thyroid carcinoma (RI refractory TC). But it provides an alternative treatment strategy to the metastatic RI refractory TCs (which have either lost the ability to concentrate iodine or were never iodine avid). Hence, larger studies are needed to define PSMA's role in the management of RI refractory TCs.

## Conclusion

MPMs are very rare, but their prevalence has been increasing with better treatment and improved survivable rates among cancer patients. Nuclear medicine, with its vast array of radiotracers, can be extremely useful in early detection of such cases, potentially reducing the need for multiple biopsies.
